# The bones of the insane

**DOI:** 10.1177/0957154X13476200

**Published:** 2013-06

**Authors:** Jennifer Wallis

**Affiliations:** Queen Mary University of London

**Keywords:** Asylum attendants, General Paralysis of the Insane, osteomalacia, post-mortem, West Riding Asylum

## Abstract

This article examines alienist explanations for fracture among British asylum patients in the late nineteenth to early twentieth centuries. A series of deaths in asylums came to light in the 1870s which, in placing the blame for such incidents on asylum staff, called for a response from the psychiatric profession. This response drew upon other medical fields and employed novel pathological techniques to explain why fractures occurred among the insane, in many cases aligning bone fragility with particular forms of insanity (namely, General Paralysis of the Insane). Although such research aimed to provide a medical explanation for the ‘fracture death’, it also called into question the value of pathological research and the utility of quantitative measurement in understanding mental disease.

## Introduction

January 1870 marked the beginning of one of the most heated debates in late-Victorian psychiatry, as a number of suspicious deaths in asylums came to public attention. The most contentious was that of Rees Price, an elderly patient admitted to Carmarthen Asylum who died shortly after admission. A post-mortem found eight broken ribs, and it was alleged that Price had received no proper examination on admission, or any special attention when he began to exhibit breathing difficulties. It soon transpired that in October 1869 Santa Nistri had died at Hanwell Asylum with eight broken ribs and a broken breastbone found at post-mortem; a few days after the Price scandal, William Wilson at Lancaster was reported to have twelve broken ribs when he died (Anon., 1870a; Anon., 1870b).

On 17 Jan. 1870, a letter appeared in the *Pall Mall Gazette* in response to the news (Reade, 1870). It was penned by Charles Reade, author of *Hard Cash* (1863), a novel in which one character finds himself in an asylum at the mercy of sadistic attendants. Reade claimed to be able to cast light on the situation and cited research he had undertaken when writing *Hard Cash*, arguing it was common practice for attendants to ‘[walk] up and down [patients] on [their] knees’ in order to subdue them. While the *Pall Mall Gazette* took up the mistreatment of the asylum population as its own personal crusade, letters from former patients further propagated the notion of attendants ‘kneeling’ on patients. Criticism also spread beyond popular newspapers into the medical press. An especially damning piece appeared in the *British Medical Journal* in October, listing four instances of broken ribs besides Price, Nistri and Wilson, and noting that ‘rib-crushing’ was ‘the favourite … mode in which lunatics [were] hurried out of existence’ (Anon., 1870c: 441).^1^ Although asylum attendants received the brunt of the criticism, blame was also extended to Superintendents for not surveying their staff properly (Anon., 1870d: 1403) and to the Commissioners in Lunacy whose tardiness in investigating deaths was interrogated (Anon., 1870a: 251).

The question of who was ultimately at fault for patients’ broken ribs did not yield a clear answer. At the heart of the issue was a much more philosophical problem about who was to blame for the deaths of those who were not like ‘normal’ people. Throughout the debate, the asylum patients’ inability to look after themselves was repeatedly emphasized, with many deaths said to have occurred as a result of falls or other accidents. The excitement, delusion or physical infirmity of the asylum patient was made to account for a wide range of events within the asylum walls, so that apparently inexplicable injuries could be rationalized by attributing them to the patient’s dysfunctional behaviour. Though an accident traditionally implied no human agency, changing understandings of the accident blurred the boundaries between accident and intentional act, so that ‘accidents’ coalesced to form a body of cases explained in the press as events neatly attributable to a corrupt asylum system.^2^ Far from being apathetic about the fate of the asylum patient, there was a distinct thread of humanitarian concern running through many of these press accounts, suggesting the humanitarian narrative described by Laqueur. He identified ‘an extraordinary number of hitherto untold stories of human suffering’ (Laqueur, 1989: 190) disseminated in the nineteenth century (with particular focus on the poor) via Blue Books of parliamentary inquiries, which filtered into the mainstream press. This process of inquiry was ‘explicitly tied to sympathy for the plight of strangers’ based on the common experience of the body (p. 191). Newspaper articles certainly capitalized on the physical details of cases, describing how many ribs were broken and by what means injuries were believed to have been sustained. Ribs became, then, corporeal objects that provided a way into wider debates about the efficacy of the asylum system.

In many ways, the broken rib scandal was reminiscent of the concern for chloroform deaths (patients dying while under sedation), which had garnered significant public interest in the 1840s and 1850s. Like chloroform death, the broken rib phenomenon, though not particularly common, ‘was recurrent and afforded no clear causal explanation’, and – while a criticism of medical care was obvious – a degree of culpability was also placed on the patients themselves, with death partly explained as a consequence of their predisposition (Burney, 2000: 139). This concern for patient autonomy readily extended to asylum patients who were wholly dependent on staff for their day-to-day welfare. As one commentator noted, ‘a fracture which would pass unnoticed in a private family [became] most properly the subject of public judicial enquiry when it [occurred] in the inmate of a lunatic asylum’ (Ormerod, 1871: 576), suggesting that asylum staff bore responsibility for their patients much as a parent might for their children.

Many press features hinted that the news items reaching them were merely the tip of the iceberg: elsewhere ‘all [went] on merrily; lunatics’ ribs [got] broken somehow, or [broke] by themselves; the fact of death [was] notified to friends, to whom the news [came] as a relief [and] quiet funerals [took] place’ (Anon., 1882: 381). Official statistics suggested otherwise: the Blue Book of 1896 recorded 7,182 deaths in English and Welsh asylums, 11 of which were a result of fractures or dislocations (Briscoe, 1898: 1677). The *Journal of Mental Science* declared that fractured ribs accounted for just one in every 2,000 deaths, or one in 1,000 where post-mortems took place (Anon., 1882: 383). Some speculated that fractures were less common in the asylum due to extra precautionary measures (Anon., 1871: 634), while others cited increased inspection as the reason for deaths coming to public attention (Anon., 1870e: 140–1). The recording of accidental injury was indeed increasing at this time; the role of the Commissioners in Lunacy was mirrored by the factory inspector of the 1890s, and Cooter (1993: 87) has charted the rise in accidents recorded by the London Hospital from 5,503 in 1842 to 18,501 in 1908. Uncovering the truth of such statistics is a difficult exercise, with their production rooted in an era when government regulation and measurement were self-consciously extending their reach; thus, some commentators refer to the ‘social construction’ of the accident, with a greater number identified in a greater number of locations (e.g. Bartrip and Fenn, 1990). Similarly, just as we may read sensational accounts of broken ribs as evidence of a widespread phenomenon, we may also read the reports as exaggerations of a few isolated cases – as Edward Baines noted of the cotton industry in the 1830s, where a few injuries led to condemnation of the industry as a whole (Bartrip, 2002: 26). A great deal of work has already been done which examines our interpretation of Victorian science from a modern standpoint. I do not seek here to uncover a ‘true’ version of events – whether broken ribs were or were not a consequence of violence by attendants – or to evaluate the robustness of asylum research. Rather, I aim to examine the discourse surrounding the phenomenon: how broken bones in the asylum were conceptualized and explained, and their relevance within late Victorian psychiatry more broadly.

Although broken ribs and bones were reported in many asylums – both in Britain and elsewhere – the key examples that I use in this article come from the West Riding Pauper Lunatic Asylum in Wakefield. As will become clear, the West Riding Asylum is particularly relevant to the debate about asylum fractures by virtue of the experiments undertaken by its staff in the later nineteenth century. Opened in 1818, this Asylum played a key role in nineteenth-century psychiatry, particularly from the 1870s onwards. James (later Sir James) Crichton-Browne, Superintendent in 1866–76, was instrumental in pushing the asylum in a more self-consciously scientific direction, appointing a pathologist, setting up a laboratory, and editing six volumes of the *West Riding Lunatic Asylum Medical Reports* (1871–76), containing original research by Wakefield’s staff and visiting researchers. Successive Superintendents Herbert Major (1876–84) and William Bevan Lewis (1884–1910) continued Crichton-Browne’s endeavours; the latter was especially active in pursuing histological research and oversaw the opening of an acute hospital in 1900.

## Identifying the problem: the fracture death and the vulnerable patient

Discussions of broken ribs centred upon the evidence offered by the body of the patient, usually at post-mortem or coroner’s inquest. This was often the first point at which such injuries became apparent or, as the *Pall Mall Gazette* put it, the point at which ‘the old, terrible story [of] half a dozen … crushed-in and broken ribs’ was disclosed (Anon., 1870f). At post-mortem, the body had the power to reveal ‘what really happened’ (Laqueur, 1989: 195), and in late nineteenth-century alienism the procedure was peculiarly important to the study of mental disease due to its ability to uncover what might be concealed during life. ‘Insanity masks and modifies the symptoms of bodily disease,’ noted Crichton-Browne, ‘so that pneumonia may sometimes be found after death when its presence had not been betrayed during life … And it is the same with other diseases and with injuries.’^3^ The post-mortem was crucial in understanding how mental disease could affect the body by collecting together a number of specimens in order to identify patterns, as well as in clarifying doubts about death (Andrews, 2012: 9).

Suspicious deaths became the subjects of coroners’ inquests; after the 1836 Births and Deaths Registration Act, it became a legal requirement to report all deaths, and the 1862 Lunatics Amendment Act required all asylum deaths to be reported to both the local coroner and the Commissioners. At the West Riding it was standard practice for the coroner to hold an inquest, not only in cases of suspicious deaths, but for deaths occurring a few days after admission.^4^ The coroner’s warrant books for the West Riding spanning 1834–79 contain 384 cases, 44 of which mention ‘fracture’ as a sole or ‘accelerating’ cause of death.^5^ One of these cases, Henry D., predates the scandals of 1870 but highlights how the key elements of the broken-rib case were already present a few years earlier. Henry was admitted to the asylum on 24 April 1863 from the Wakefield Workhouse where he had been discharged after a short prison stay. On 27 May, a lengthy entry in the casebook recorded:

This was a case of Dementia with excitement and also suffering from General Paralysis. He was in feeble bodily health and reduced in flesh … He was observed [at] admission to have slight *yellowish-black bruises or discoloration over the left side of the sternum* & over the right & left lumbar regions. These bruises were considered by the House Surgeon at the time to be trivial & as the patient was restless & did not complain nor appear to be in pain, no special examination of the chest was made until a fortnight (or rather more) after admission. This patient continued restless & mischievous, wet & dirty in his habits and frequently undressing himself or appropriating the clothes of other patients at the same time muttering to himself incoherently. He was never at any time violent. He *occupied a single room* at night and would *move the bed-stead too* [*sic*] *&; fro & climb up upon it* to the ventilator above the door. The bedstead was consequently removed and owing to the padded room being occupied he slept for a few nights upon a mattress placed upon the floor. … [He] continued without any noticeable change until Saturday the 16th inst. on the morning of which day, he broke a pane in a glass door leading into the Airing Court & tried to get through it. He was reported to the House Surgeon ‘as not so well’ and on examining him in bed he was found to have a broken rib on the right side & to be suffering from Pleurisy. He was placed under treatment but gradually became worse and died on May 19th.^6^

In this extract, the words printed in italics are underlined in blue pencil in the record, suggesting that the pertinent points of the case were highlighted after death when the case became of legal concern. The post-mortem found seven fractured ribs. The patient was free from the bruises described above, and the appearance of the ribs apparently indicated that the fractures ‘had occurred not less than 2 weeks before death but might have been of 6 or 8 weeks standing.’^7^ Henry D.’s case constituted a classic case of rib fracture: he was restless (pushing his bedstead around the room) and incurred fractures that were the possible result of his own actions (breaking through a glass door).

It is clear from examining Wakefield’s records that by no means all cases of fracture resulting in, or occurring close to the event of, death became the subject of coroner’s inquest, however. Thomas T., aged 69, was found to have three broken ribs at post-mortem, yet the coroner attributed his death to a combination of bronchitis, pleurisy and erysipelas of the arm. Explaining his decision, he said: ‘As to the fractured ribs it was evident they were not of quite recent date and it was thought probable that they had occurred by one or more falls, which could hardly be prevented.’^8^ This tendency to dismiss some fractures as contributory causes of death according to their circumstances was also evident in the case of Widdop P., a 38-year-old man diagnosed with general paralysis who died 11 days after admission:

A broken rib was found after death and an enquiry held accordingly. From the probable date of the fracture (it was but quite recent) and from the fact that previous to being brought to the Asylum, the patient had fallen headlong down stairs, it was decided by the Jury that the accident had occurred to the patient previous to admission, the Attendants being exonerated from all blame or suspicions.^9^

Evidently the coroner, rather than judging deaths solely on the basis of physical evidence, might also address the circumstances surrounding death; this holistic view, however, was not always met with satisfaction.^10^ In 1868 the Asylum and the coroner attracted criticism in the local press due to the verdict returned by two inquests in which fractures were present: death was ruled in both cases to be due to ‘Natural Causes’ and the fractures present deemed ‘unassociated’.^11^ The *Wakefield Express* alluded to the cases in an article entitled ‘Fractured ribs in asylums’,^12^ suggesting that the deaths merited serious investigation. James Crichton-Browne condemned the piece for ‘[creating] the impression that a deliberate homicide was perpetuated and hushed up at this Asylum’.^13^

When the registration of cause of death began in 1838, accidents were typically grouped together with other ‘violent’ deaths, but Green (1997) argues that as the century progressed and birth and death statistics became more sophisticated, deaths involving violence (including accidents) began to be classified along lines of culpability and more thoroughly investigated. In the case of asylum deaths, the general consensus was that the onus lay with the attendant, who was responsible for the patient’s welfare at the time of his or her accident and had not exercised proper attention. As the perils of modern life intersected with naturalistic thinking, ‘people thought that accidents took place when someone who should have been able to control events did things wrong’ (Burnham, 2009: 221). Thus, stories abounded of accidents that had befallen patients when their attendants had momentarily left the room or turned their backs: a scalding in a bath, a tumble down some stairs, a laceration from a window pane.

Accidents are also not as random as their definition might suggest (Campbell R, 1997; Davidson and Townsend, 1982: 126–8). They tend to occur towards the lower end of the socio-economic scale and – just as occupational diseases or deficiency diseases such as rickets ‘had a strong tendency to social class specificity in their choice of victims’ (Bartrip, 2002: 2) – so too could the broken ribs of asylum patients be viewed as an ‘epidemic by instalment’ (p. 2) affecting a particular social group. It is understandable that the correlation of injury/asylum was explained by placing blame on asylum attendants, but the problem could also be seen from another perspective: that the insane were genuinely predisposed to fracture or incidents which put them at increased risk of sustaining injury.

## ‘A distinct pathological entity’?^14^

As popular interest grew, the medical press witnessed a corresponding increase in articles detailing fractures in the insane, while works on bone disease frequently included short sections dedicated to the topic.^15^ The discourse recalls that identified by Sammet (2007) on haematoma auris in early nineteenth-century Germany: asylum staff, dismissing the possibility that haematomas of the ear were caused by attendant violence, explained them as the result of an underlying condition in patients suffering ‘inflammatory irritation of the meninges’ (Sammet, 2007: 293). The diagnosis was visual, at first with the naked eye then via microscopical investigation. While Sammet (2007: 298) concludes that ‘[a]lienists’ obsessive exploration of an unimportant lesion showed their concern about their inability to ever become the real rulers’ of their institutions, the problem of broken ribs in the asylum differed from that of haematoma auris in one important respect: fractures were not trivial, but often proved fatal and propelled the asylum directly into the spotlight.

One condition predominated in these discussions of fracture among the asylum population: General Paralysis of the Insane (GPI). Now believed to refer to tertiary syphilis, GPI was a familiar sight to nineteenth-century superintendents, characterized by grandiose delusions and various reflex disturbances.^16^ It was a fatal condition: many patients diagnosed with GPI died within a few months, sometimes weeks, of admission. W.H.O. Sankey (a Superintendent at Hanwell, then in private practice) had noticed that fractures found at post-mortem tended to be in those patients who were male and recent admissions. The extent of the fractures was beyond what would be expected as the result of a simple fall; nor, he surmised, could so many breaks occur as a result of attendants ‘kneeling’ on patients. Instead, Sankey attributed such injuries to the impaired reflexes and dulled sensations of GPI patients who ‘[threw] themselves about with reckless violence’ (Anon., 1870e: 138); this, he said, explained the preponderance of male cases (GPI was more often diagnosed in men) and recent admissions (admitted at the height of characteristic paralytic violence). Sankey highlighted the key elements of GPI that led to injury: impaired sensations, great excitement or violence, and muscular weakness leading to clumsiness (see also Brown and Rogers, 1870: 96).^17^ In West Riding patient Thomas S., both a lack of reaction to injury and excitability were evident, his case having been assessed at admission as one of acute mania and suspected GPI.^18^ His death was preceded by the discovery of several fractured ribs, attributed to an incident two days previously:

… the patient … having been taken to a seat in the dayroom of his ward, suddenly got up ran down the gallery and kicking over a bucket which was in use fell headlong upon it. The patient did not seem hurt at the time and ate a good breakfast afterwards so that although the accident was reported by the attendant it did not attract special attention at the time.^19^

The importance of GPI in Thomas S.’s death was clear in the coroner’s verdict: ‘accidentally falling over a slop pail in the gallery of no. 18 ward, and thereby fracturing his ribs and causing pleurisy – he being at the time in an advanced stage of general paralysis’.^20^ The characteristic excitement of GPI led to Thomas’s accident, but the condition also complicated subsequent treatment as his diminished sensations allowed him to ‘[eat] a good breakfast afterwards’ and give no cause for concern. Lack of complaint from patients was a common theme: Walter M. ‘whilst at work … had a severe fall but said nothing about it[,] went about as usual and made no complaint of injury until, attention being attracted by his delicate appearance he was examined physically’.^21^ At this point, fractures were found which Walter attributed to a blow while working, but which he dismissed as painless. In contrast to the general hospital patient, it was the asylum patient’s non-response to accidents that marked them out as especially problematic. The GPI patients also had a tendency to place themselves in dangerous situations: they held ‘very exalted notions of their own power and ability, and a strong propensity to order and direct every one else … combined with great muscular weakness, diminished sensibility to pain, and inability to protect themselves’ leading to quarrels with others where they were ‘at a disadvantage’ (Anon., 1870g: 254). Even left alone, their restlessness and lack of physical control might impact on their physical well-being, with falls out of bed often resulting in injury.

Not all cases of fracture could be traced back to an accident or violent incident, however, leading some to look to the asylum environment itself as a factor in bone disease that increased the tendency to fracture. ‘I do not think that asylum life [produces bone] disease’, wrote Scottish alienist William Carmichael M’Intosh, ‘but certainly I think it would aggravate the tendency’ (M’Intosh, 1862: 150). If patients spent their days sitting in wards or confined to bed, it was hardly surprising that their physical health would suffer. The poor state of many patients also militated against their recovery from relatively minor injuries. Commenting on the death of one patient after a leg fracture, Herbert Major noted: ‘In a younger and healthier subject than the patient was, the injury would not probably have been attended with any serious consequences but in the debilitated, unhealthy constitutional state in which he was … it brought about a fatal issue’.^22^

The argument that environment could affect the fabric of the body, coupled with a desire to link mental disease to underlying somatic disorders, gave alienists reason for optimism. The discovery that weakened bones were linked to GPI would not only go some way towards absolving asylum staff of the charges made against them, but demonstrate the value of asylum science to the study of mental disease, and to medicine more generally. It was at post-mortem that alienists found some of the most striking evidence of bone disease in their patients, suggesting that the physical behaviour of the patient may not be the only explanation for fracture. George J. Hearder, Carmarthen Superintendent, found nine out of his 20 post-mortems revealed ribs in a ‘diseased state’ (Hearder, 1870: 566), mirroring the findings of investigators both inside and outside the asylum who graphically described the unusual appearance of the bones under study. They could be snapped between two fingers, were ‘soft and boggy’ (Mercer, 1874: 541), ‘like wet leather’ (M’Intosh, 1862: 146) or ‘sponge soaked in fat’ (Pedler, 1871: 165–6), and when cut exuded ‘a thick bloody fluid’.^23^ Some described being able to tie bones in a knot at post-mortem due to their incredible flexibility (Macnamara, 1878: 222–3), and their anomalous appearance might be evident for years to come – remaining dark and rotten when preserved (Ormerod, 1871: 572).

These observations typically led to a post-mortem diagnosis of mollities ossium, or osteomalacia –an abnormal softening of the bone. Outside an asylum context, osteomalacia was most often seen in women and the elderly, manifested via symptoms of bone pain and a tendency to fracture easily; in extreme cases, the condition could lead to distortion of the limbs and torso.^24^ The aetiology of the condition was at that time unclear. Some described it as the adult counterpart of rickets (Markoe, 1872). Others argued it was a distinct disease, involving muscle degeneration alongside skeletal abnormalities (Jones T, 1887). There was general agreement that the condition was prevalent in women, usually those who had had children. Walsh, studying four female cases at Wakefield Asylum in 1891, singled out for particular comment one woman who had the condition despite never having borne children; the presence of the disease in her case was even more extraordinary because she was the only one of the four still living (Walsh, 1891: 170). The condition’s presence in the elderly, another group in which it seemed especially common, was explained as one of the natural processes accompanying senility, and a consequence of unhygienic surroundings and insufficient diet.

That mollities ossium might be seen in a range of demographic groups thus opened up the possibility of viewing the condition as coincident with a generally deficient bodily system. Indeed, Markoe’s *Treatise on Diseases of the Bones* (1872: 55) had described rickets not as bone disease, but a symptom of wider imperfection. In a climate that sought to classify bodies according to their differences, and in view of the fact that the poor physical state of many patients was often painfully evident, it was logical to add the insane to this list of deficient bodies. Thomas Smith Clouston, writing on phthisical insanity, described the bodies of the insane as ‘fertile seed-beds’ for bacteria, arguing that any disturbance in brain function would sooner or later yield trophic changes (Clouston, 1892/2005: 482). In the same vein, Coutts (1887) correlated rickets with epilepsy, reasoning that osseous changes in the growing body could be detrimental to long-term mental health. J.C. Brown (Liverpool Royal Infirmary) and T.L. Rogers (Rainhill Superintendent), noting that the bones of a general paralytic patient possessed a ‘general appearance … so unlike that of the ribs of healthy adults’ (Brown and Rogers, 1870: 89), found their composition not dissimilar to the bones of osteomalacia. Similarly, clinical assistant George Pedler’s analysis of the bones of 540 patients at Wakefield Asylum declared only half of the average insane patient’s bone to consist of ‘true bone’, the rest having been replaced by ‘oily and fatty matters’ (Pedler, 1871: 166).

At Wakefield in 1871, it was reported that four women had died of mollities ossium.^25^ It was not uncommon to cite mollities ossium as a cause of death: it was understood as a progressive condition in which ‘nothing [could] be accomplished but the palliation of suffering’ (Markoe, 1872: 85). Orthopaedic specialists such as J. Jackson Clarke (1899: 34) dismissed the condition as one worthy of their investigation for precisely this reason as it offered little scope for medical intervention. Many alienists, however, could ill afford to ignore the issue of fracture among their patients, and post-mortem evidence offered an ideal illustration of the atypical pathology of the insane. It was not enough, however, simply to reiterate examples from personal experience to prove a tendency to bone breakage; indeed, the retrospective tone of many examples might merely fuel public suspicion. If alienists were to demonstrate conclusively that fragile bones were a common phenomenon in insanity, they would have to offer some concrete proof.

## Quantifying bone fragility

When investigating fracture, it was the strength of the bone – rather than its appearance – that had most immediate relevance. Clouston had tested the bearing weight of the ribs of the insane in 1870 (Anon., 1871: 634), and by the 1880s Joseph Wiglesworth offered perhaps the most complete account of the issue. The entry ‘Bone degeneration in the insane’ in Tuke’s *Dictionary of Psychological Medicine* (1892: 143–5) was written by Wiglesworth. Here, he summarized contemporary knowledge about bone disease, noting that there was ‘nothing remarkable in the circumstance that the … failure of nutrition [seen in the insane and evident in brittle hair and harsh skin] should extend to the nervous system’. Wiglesworth admitted that nutritive failure was not confined to the insane, but argued that wasting diseases affecting the bodily fabric were more common among them. He concluded that lunatics’ ribs were healthy in a minority of cases and that most cases of fracture could be attributed to old age, but that in around 10 per cent there were clear lesions causing abnormal fragility. This 10 per cent would be the main topic of investigation over the next few years. Some of the most thorough attempts to quantify bone strength were made at the West Riding Asylum in the 1890s, using large patient populations and making the breaking strain of ribs a standard subject of post-mortem examination.

Although Pedler had tested the breaking strain of ribs at Wakefield for his article in the *West Riding Lunatic Asylum Medical Reports*, he had neglected to give any detailed information about his methods (Pedler, 1871). Reviewing the *Reports*, an anonymous writer in the *Journal of Mental Science* doubted the utility of such tests, and in order to prove the point conducted experiments (also unexplained) ‘showing that the test by weight, etc., was not reliable’ (Anon., 1872: 561). Theo Hyslop, reflecting on his time as Clinical Assistant at Wakefield Asylum, said that he had undertaken experiments into breaking strain using ‘an ordinary concrete testing machine’ (Campbell AW, 1895a: 273). In the 1890s, however, that investigation became more systematic, aided by a device invented and distributed by Charles Mercier. The fullest description of this innovation appeared in Alfred Campbell’s ‘The breaking strain of the ribs of the insane: an analysis of a series of fifty-eight cases tested with an instrument specially devised by Dr. C.H. Mercier’. He briefly described an instrument sent to him by Mercier in 1893 ‘for the purpose of automatically registering the breaking strain of the human rib’ (Campbell AW, 1895a: 254). To do this, one ‘extract[ed] a certain length of the eighth pair of ribs, and test[ed] the breaking strain of one of these lengths against the convexity, of the other against the concavity’; an inch of bone was also taken for microscopical examination (p. 255). Mercier described the instrument thus: ‘It had a stirrup at one end and a screw at the other, and between these was a spring which registered the number of pounds pressure exerted. The bone … was put through the stirrup resting on the fork of the machine; the screw was then turned till the rib broke’ (p. 272). He had sent the instrument to several asylums as well as larger London hospitals, suggesting that he had to some extent mass-produced it (p. 272).

Why did Mercier take this step in the early 1890s when, as Tuke’s *Dictionary* asserted, occurrences of broken ribs were believed to have become ‘infrequent’ (Tuke, 1892: 1094)? Also, by this time the excitement of the mainstream press had died down somewhat. Even in the 1880s, it was noted that fewer cases of fracture were coming to light, possibly due to greater care being taken by attendants (Wiglesworth, 1883: 630). Mercier, however, was determined that a ‘full series of experiments’ should be made and the results collated so that alienists ‘would be able to go before a coroner with a good face’ (Campbell AW, 1895a: 271). If we look at wider developments in the 1890s, it is evident that the introduction of Mercier’s instrument reflected more general concerns. Firstly, regulation of the workplace, encapsulated in legislation such as the 1895 Factory Act, was a subject with interest far beyond the factory walls. Havelock (1899) pondered the 1897 Workmen’s Compensation Act’s relevance to asylums, and whether an asylum where patients took part in labour could be considered financially liable for accidents. Clearly, the Act’s model of employer liability could be extended to a variety of contexts, the relationship between patient and asylum staff mirroring that between worker and employer. Occupational health literature emphasized the effect of long-term illness on industrial efficiency, and by the end of the century the developing field of orthopaedics was beginning to intervene in ‘critical locations’ such as workhouses in an attempt to ‘fix’ the anomalous bodies of the poor; the dwelling places of the labouring classes were becoming less private, subject to both philanthropic and governmental inspection (Cooter, 1993: 9). Secondly, the Lunacy Act of 1890 updated and expanded the 1845 Act. The earlier Act had stipulated that abuse of patients by asylum staff was a chargeable misdemeanour, and the Lunatics Amendment Act of 1853 had required asylums to make known to the Commissioners any cases of dismissal for neglect or cruelty. In 1890 prosecution became a more distinct possibility in such cases and restraint more closely regulated, with mechanical restraint recorded in a central register. The concerns of the 1890 Act bring us to a third, broader point: a general suspicion of medicine. A resurgence of interest in anaesthetic deaths in the 1890s had partly contributed to the establishment of the Society for the Protection of Hospital Patients in 1897, for example (Burney, 2000: 149). As the professional was himself pathologized as a slightly sinister figure, both pre- and post-mortem procedures had to be absolutely necessary, with the overriding concern being the benefit to patients. In the asylum, the post-mortem was partly rationalized as a deterrent, preventing attendants from ‘ill-using patients, as injuries inflicted upon them [were] sure to be detected’ (Adam, 1884: 362).

Bearing in mind the purposes of post-mortem examination, what did investigations into breaking strain find? Campbell’s first paper was confident in identifying an average breaking strain of 44.8 lbs convex and 44.4 lbs concave in his male GPI subjects compared with 62 lbs and 65 lbs, respectively, in a healthy adult male (Campbell AW, 1895a: 256). His second paper on the subject, published only a few months later, was more hesitant and cast doubt on the link between fragile bones and insanity: ‘The difference between the average breaking strain of the ribs of the insane and that of the ribs of persons free from mental disease is not so great as one would anticipate’ (Campbell AW, 1895b: 776). In this larger sample of 58 Rainhill patients and 50 Royal Southern Hospital patients, Campbell found very little difference between the breaking strain of the male asylum patient (41 lbs convex, 42 lbs concave) and that of the male general hospital patient (43 lbs convex, 43 lbs concave). He theorized that wasting diseases had greater influence on bone structure than mental afflictions, though of course GPI had a place on both sides of the argument. Campbell (1895b) was also forced to admit the existence of anomalies, making any concrete conclusions difficult: for example, two sane female patients from the general hospital had exhibited a breaking strain as low as five pounds.

The inconclusive nature of Campbell’s results did not, however, make the measurement of breaking strain entirely redundant. Mercier reported that, apart from Campbell, he had received no reply from any of the asylums who received his instrument, with the exception of William Lloyd Andriezen at Wakefield (Campbell AW, 1895a: 272). Andriezen had joined Wakefield Asylum’s staff as a medical officer in 1893 at the young age of 26.^26^ Discussing Campbell’s first paper, Andriezen reported that he had used Mercier’s instrument in 122 Wakefield post-mortems (Campbell AW, 1895a: 273). An examination of the asylum’s post-mortem reports shows that breaking strain was systematically recorded from 30 September 1895. At the beginning of that month, pre-printed certificates appeared with spaces for the name, date of death, etc., and also contained a pointed reminder of the details to be included in the post-mortem record: ‘The following particulars are Statutory:- Condition – External Appearances – Bedsores – Head – Thorax – Describe *ribs* in every case – Abdomen – Weights – Microscopic Appearances and Special Notes.’^27^ (emphasis in original). There remained in the 1890s, then, an obvious concern for the state of patients’ ribs, one that loomed large enough to influence record-keeping. The post-mortem’s dual purpose – as a means of discovering bodily disease and proving the good treatment of patients – is evident in the need for ‘Microscopic Appearances’ alongside the presence of bedsores.

Although breaking strain was being recorded on a regular basis by Andriezen (and other medical officers), he never published the findings of the tests he alluded to during the discussion of Campbell’s paper, but Francis Simpson rectified this lack of data in his book, *The Pathological Statistics of Insanity* (1900). He had come to Wakefield in 1894 and demonstrated such enthusiasm for statistics that by 1896 he was moved to request a ‘calculating machine’ to deal with the large numbers involved in his ‘tabulat[ion] of the results of post mortem’.^28^ *The Pathological Statistics of Insanity* contained the results of this data collection, listing the weights of 398 brains and their constituent parts. As brains were Simpson’s self-confessed focus, it is noteworthy that the only other quantitative data in the book was the breaking strain of the ribs. He tabulated breaking strain both by gender and form of insanity based on measurements obtained with Mercier’s instrument; like Campbell, he questioned the link between bone fragility and general paralysis, and instead emphasized the effects of age.

By this time, the GPI/fragile bone relationship seemed to be in some doubt as a result of pathological examination. If bone fragility was a consequence of ageing rather than mental disease, alienist claims for expertise became much less relevant. Despite concern for fractures in coroner’s inquests and physical examinations, they were unable to suggest much beyond taking special care of ‘at risk’ patients. Other technical innovations in the alienist field had, like Mercier’s instrument, arisen out of concerns for the mistreatment of patients; Sammet (2006) has examined the development of rectal feeding as an alternative to oral force-feeding in Germany, for example. Technologies like rectal feeding had clear beneficial effect as the emaciated patient grew in strength. Pathological technologies, however, were of no practical benefit to the deceased patient whose body they acted upon: to many commentators investigations into breaking strain merely added insult to literal injury.

The skill of the individuals conducting such tests also came under fire, with general practitioners criticizing the supposedly amateur post-mortems in asylums. Pathology did not easily lend itself to professionalization when a pathologist’s tools might consist of ‘ham knives’ and ‘butcher’s saws’ (Burney, 2000: 120), and there was a tendency to view the asylum pathologist – like the asylum attendant – as under-qualified for his position (one can imagine the popular response to a doctor conducting experiments with a ‘concrete testing machine’). Bone specialist Charles Macnamara, though willing to accept the existence of an inherent condition causing fragility, was doubtful of the value of breaking strain tests, saying they were ‘hardly sufficient to convince you that the ribs of insane patients [were] at times diseased to such an extent as to render them extremely brittle’ (Macnamara, 1878: 230). He questioned the skill of the asylum pathologist in view of the fact that his own experiments had found no such pathological changes, and argued that such investigations should only be undertaken by those pathologists with special knowledge of the osseous system.

Despite sceptical reactions to breaking strain experiments, their findings did feed into practical changes. These brought the debate full circle, once again giving asylum attendants the responsibility for preventing injury. While the duties of the attendant were not clearly defined in the early nineteenth century, the second half of the century saw a concerted effort to mould attendants into an efficient and effective workforce, dedicated to the growing field of psychiatry (Nolan, 1993). In 1890 the Medico-Psychological Association (MPA) adopted the *Report on the Training of Nurses* (Digby, 1985: 165), and in 1891 the Certificate in Attendance and Nursing upon Insane Persons was introduced; by 1899 over 500 of these were being granted each year (Crammer, 1990: 99). Official qualification complemented other moves towards a better-regulated occupation, such as the introduction of the MPA’s *Handbook for the Instruction of Attendants on the Insane* (1886). The *Handbook*’s contents ranged from an overview of the Lunacy Acts, to good practice in matters such as ward ventilation. The issue of restraint was also prominent, with readers advised never to place their knees on the body of the patient should ‘a struggle be unavoidable’ (MPA, 1886: 126). The spectre of broken ribs appeared more explicitly in Mercier’s *The Attendant’s Companion* (1898: 31): ‘under no circumstances whatever should a patient be knelt on’, he warned (in bold text), as this could lead to broken ribs and breastbones. The fragile bone theory was made explicit, with Mercier (1898: 25) reminding readers that:

… very many of the inmates of asylums are advanced in years, and the bones of old people are easily broken. It must also be remembered that the bones of some patients, and especially of those suffering from general paralysis become unusually brittle … and a knock or a fall which would be of no consequence whatever to a young person in ordinary health, may readily break some of the bones of such patients, and produce very serious injuries.

The MPA’s *Handbook* (1886: 57) instructed attendants to report to a medical officer any complaints of pain or a ‘shrinking away’ from contact that might suggest a fracture, as well as any bruises or other abrasions noticed during dressing and bathing. Mercier (1898: 57) emphasized that only a medical officer could provide a definitive diagnosis, reflecting the view that for asylum attendants ‘a little learning [was] a dangerous thing’ (Bevan Lewis, 1907: 124). When the fifth edition of the MPA’s *Handbook* was published in 1908, it was criticized for its increasing focus on anatomy rather than psychiatry (Crammer, 1990: 99). There was a sense that, despite the introduction of official certification and training, asylum staff remained if not morally, then at least intellectually, inferior.

## Assessing the fragile bone theory

By the early twentieth century, Wakefield Asylum’s post-mortem records displayed a distinct lack of concern for the breaking strain of patient’s bones. Despite the form providing a pre-printed line for ‘Ribs’, there was no meticulous charting of breaking strain; instead, vague statements were used such as ‘Rather Soft’ and ‘Softish’.^29^ That the post-mortem records were kept in this fashion suggests that breaking strain was considered less useful as a pathological fact, but also raises the possibility that a normal standard of rib strength was generally agreed upon and needed no detailed elaboration (indeed, one record noted ‘Ribs rather softer than normal’^30^).

At the same time, William Maule Smith presented ‘On the nature of fragilitas ossium in the insane’ at the annual meeting of the British Medical Association, describing the results of his analysis of 200 post-mortems at Wakefield (Smith WM, 1903). He had not used Mercier’s instrument, preferring to rely on tacit knowledge: his ‘conclusions rested on the ease with which fracture was produced by digital compression’ (Smith, 1903: 824). His findings confirmed much of what Simpson had demonstrated, casting doubt on the idea that bone fragility was ‘a marked pathological condition in … general paralysis’. Discussing Smith’s paper, and his later lantern-slide demonstration of microscopic specimens (see Anon., 1904), familiar criticisms were voiced. Mercier noted, no doubt with a hint of mischief, that Smith’s observations on breaking bones with a finger ‘scarcely carried to an outsider the same conviction that could be produced by a numerical comparison’ (Smith, 1903: 827), while others cautioned against placing too much faith in pathological observations (Anon., 1904: 189).

As well as technical quibbles, the precise motivation for experiments into breaking strain was frequently unclear. Many articles gave the sense that individuals were explaining any doubtful incidents in their institutions before they were brought to light by a sensationalistic press. Such confessions, however, did not overcome the gulf that existed between alienism and medicine ‘proper’, and the public. Conflicting professional knowledge was often evident in court cases investigating fracture deaths; Joseph Workman (Superintendent of Canada’s Toronto asylum) was critical of a case in which it had been argued that multiple ribs could not possibly be broken without some pain, but in which no testimony as to the diminished sensations common to general paralysis had been heard (Lindsay, 1870: 419). While the insanity defence might absolve an individual of responsibility, the insanity-as-pathology defence at the heart of the broken rib debate rarely served to exonerate asylum officials from blame.

Yet the fragile-boned asylum patient was clearly an appealing explanatory model. Broken ribs became something of a self-fulfilling prophecy: Ormerod (1871: 571) noted that ‘the more attention has been called to [them], the more frequent does the occurrence seem to become’, suggesting that to some degree broken ribs fulfilled what Kanaan and Wessely (2010: 68) have called a ‘diagnostic need’. In the case of asylum fractures, a diagnostic need could certainly be deduced in the face of public scrutiny, but the influence of a drive towards pathological research must also be considered. Fractures may have thrown the alienist profession into disrepute but they could also, as the subject of detailed investigation, furnish new knowledge about mental disease and speak directly to alienist attempts to find a physical basis for patients’ conditions. The idea that the insane were peculiarly prone to bone disease was one that fitted logically alongside wider theories about both disease susceptibility and the general health of the asylum patient.

However, the response to the theory complicates the conclusion of Burney (2000: 164) that (in the case of the anaesthetic death) ‘[p]ost mortem accounts of preexistent [*sic*] organic dysfunction at once confirmed medicine’s capacity to serve as the master narrative of death causation, even when forced to take itself as the subject of inquiry, and provided a reading compatible with the needs of public inquiry.’. When investigating fracture deaths in which the asylum itself was certainly a ‘subject of inquiry’, explanations that emphasized the peculiar pathology of the insane patient rarely received a favourable reception from the public, or the majority of the medical profession. ‘Naturalizing’ the accident did not necessarily disguise the social inequalities leading up to it (see Cooter and Luckin, 1997). While a broken rib could not be described as an accident proper, it was also difficult to view it in the same way as an industrial or wasting disease which developed over time – a tension sometimes illustrated by the use of the term ‘accelerating cause’ in coroners’ verdicts.

Although short-lived, the theory of bone fragility in the insane mirrors the turn-of-the-century concept of accident proneness described by Burnham (2008, 2009). He argues that, around 1900, a group emerged in occupational health discourse who ‘suffered injuries and caused damage’ on a greater scale than the majority of workers (Burnham, 2009: 19). These were people who, through no calculated effort of their own, were apt either to endanger their own safety at work or jeopardize that of others. Like the broken rib phenomenon, accident proneness raised questions of how to ‘deal with [people] who [showed] a pattern of inadvertent but sometimes dangerous destructiveness’ (Burnham, 2009: 5) and could not be held accountable for their actions. The accident prone individual and the fragile-boned insane patient were both conceptualized as ‘natural objects’ (Burnham, 2009: 221; Smith, 1981: 161) – incapable of change and only able to be saved from themselves by external intervention, whether that be safety railings around machinery for the former, the use of padded rooms for the latter, or protective clothing for both.

Despite attempts at medicalization, the broken rib theory – like accident proneness – gradually disappeared as an explanatory paradigm. While doctors and pathologists investigating bone fragility had strived to elaborate a discourse of mental disease in which responsibility was absent, their findings did not change the basic fact that the asylum patient remained an individual in need of special care. Whether fractures were the result of violence or inherent weakness, the key figure remained the attendant whose responsibilities were unchanged by the suggestion that some patients were especially liable to fracture. By pathologizing the asylum accident and drawing attention to the body of the patient, the affair inadvertently accorded the body of the patient a peculiar authority: within the dramas played out in the pages of the *Pall Mall Gazette* and in more sober narratives, the body ceased to be merely a ‘natural object’. In configuring the broken or brittle bone as worthy of particular investigation – via naked eye observations of its abnormal appearance or detailed measurement using specially-devised instruments – it was accorded a significance that impacted on the lunacy profession more widely. The post-mortem both acted upon the body of the patient and was shaped by the objects it aimed to investigate, with concerns about brittle bones entering post-mortem books and directives, case records and coroner’s warrants. Bones were capable of mediating not only asylum research, but also everyday practices and social relations, as their potentially calamitous nature was incorporated into attendant’s handbooks, and could attribute blame to their guardians in the event of injury or death. Thus, in the discourse about fragile bones, there was a dual process underway as bones became objects both constructed and constructing, demonstrating that the insane body, though often constrained, was not entirely passive even in death.

## Notes

(WYAS indicates West Yorkshire Archive Service, Wakefield)

1. This piece was later revealed to be the work of J. Thompson Dickson, whose posts included Resident Medical Superintendent at St Luke’s Hospital in London; see Anon., 1874.

2. Many historians emphasize the penchant of the Victorian press for the large-scale accident – such as the railway disaster – over the individual incident (Bartrip, 2002; Bronstein, 2008; Cooter, 1997). The intense interest in individual asylum deaths thus appears something of an anomaly within the history of the accident in nineteenth-century Britain.

3. *Report of the Committee of Visitors … 1873*: 21.

4. *Report of the Committee of Visitors … 1871*: 8.

5. WYAS C85/1117 Coroner’s warrants; C85/771 Coroner’s warrants.

6. WYAS C85/3/6/111 Male medical casebook M17: 321–2.

7. *Ibid*.: 322.

8. WYAS C85/1/13/3 Medical Director’s journals. Quarterly meeting 29 July 1880.

9. *Ibid*. Quarterly meeting 23 Oct. 1876; WYAS C85/600 Register of admissions.

10. Also see Emmerichs (2001), who casts doubt on the medical ability of many Victorian coroners.

11. WYAS C85/1/13/2 Medical Director’s journals. Quarterly meeting 29 Oct. 1868.

12. Anon. (n.d.) Fractured ribs in asylums. *Wakefield Express*; pasted into WYAS C85/1/13/2 Medical Director’s journals. Quarterly meeting 28 Apr. 1870.

13. WYAS C85/1/13/2 Medical Director’s journals. Quarterly meeting 28 Apr. 1870.

14. Briscoe, 1898: 1680.

15. There had been earlier attention to the subject, but on a less prolific scale. Wiglesworth (1883: 629) wrote that ‘Dr. Davey of Bristol … has pointed out that, as far back as 1842, he detected the presence of mollities ossium in persons dying insane’.

16. Within articles on bone fragility among the insane, there are few references to the ‘worm-eaten’ bones of tertiary syphilis despite the link contemporary writers made between GPI and bone disease. Hunt and Mann (2005: 222), however, note that bones are ‘uncommonly’ affected in venereal syphilis, with the phenomenon occurring in between 10 and 20 per cent of cases.

17. The initial response to asylum fractures is to suppose that they were largely the result of epileptic fits; Brown and Rogers (1870) commented on the rarity of such cases due to the muscular rigidity of epilepsy, which had the effect of protecting the bones from breakage.

18. WYAS C85/3/6/131 Male medical casebook M37: 505–8.

19. WYAS C85/1/13/3 Medical Director’s journals. Quarterly meeting 31 Jul. 1879.

20. WYAS C85/3/6/131 Male medical casebook M37: 508. It is notable that this patient’s condition evolved from suspected general paralysis at admission to the more definite ‘advanced state of general paralysis’ at death. Davis (2008: 113) notes how a ‘re-diagnosis [of GPI] at death’ was often made, dependent on the pathological appearances at post-mortem.

21. WYAS C85/1/13/3 Medical Director’s journals. Quarterly meeting 28 July 1881. I am grateful to Louise Hide for clarifying that, though dulled sensation was often implicated in GPI, it was not a pain-free condition, often involving severe headaches. The spinal form of the disease, tabes dorsalis, was also characterized by crippling ‘lightning’ pains.

22. WYAS C85/1/13/4 Medical Director’s journals. Quarterly meeting 25/26 Oct. 1882.

23. WYAS C85/1119 Post-mortem reports: 640.

24. An oft-cited case was that of Mme. Supiot who came under medical observation in 1752 at the age of 36: her bones were apparently so distorted as to force her legs into a position parallel to her torso. See Briscoe (1898) for two sketches of Mme. Supiot and a brief overview of her case.

25. *Report of the Committee of Visitors* … *1871*: 20–1.

26. WYAS C85/1/2/1 Minute book. Meeting 18 Jan. 1893.

27. WYAS C85/1129 Post-mortem reports.

28. WYAS C85/1/13/6 Medical Director’s journals. Quarterly meeting 17 Dec. 1896.

29. WYAS C85/1134 Post-mortem reports.

30. *Ibid*., 174.
